# Letter to Editor

**Published:** 2013-09

**Authors:** AA Haghnegahdar, P Bronoosh, H Jami

**Affiliations:** a Dept. of Oral Radiology, School of Dentistry, Shiraz University of Medical Sciences, Shiraz, Iran; b Student Research Center, School of Dentistry, Shiraz University of Medical Sciences, Shiraz, Iran

*Dear Sir*

Recently, all aspects of life have been changed & impressed by revolution of computer science. Education, as one of the most important part of life, is also affected by improvements such as Internet and E-books. Traditional books at least to some extent are slowly replaced by electronic and digital publications. These, could be produced massively in less time and lower price which means simple accessibility all around the word.

Today, instead of participating in boring classes, students can play the digital manuscripts of lectures as many times as they want at any time of the day, without getting out of the home. This eliminates the time needed for transport and its fees and risks.

In addition to potentials of multimedia used in these techniques which simplifies the process of learning, questions and tests may be applied in these digital lectures which give the user the choice to evaluate the learning process, so the student will play a more active role in process of education. Many advantages of electronic education make it desirable for most of titles. So, today, it is becoming a scale of evaluation of education institutes, i.e., the more the titles instructed by means of digital education, the higher the rank of the institute.

Digital education, in our country, although not really new, comprises a very minor part of education. Tradition classes are still the most popular method for teaching, which imparts its fees.

We have many things to do to benefit fully from potential advantage of digital education. Obviously preparing the needed hardware background is of utmost importance. Beside, lessons should be prepared in digital form and some percent of education assigned to E-learning.

Regarding the need, software, for introducing the basics of radiology, is prepared in radiology department in Shiraz dental school as a student thesis. The composition of X-ray tube, the mechanism of X-ray production, the composition of radiographic film, latent image formation and film processing are described in this software, by means of video films, animations and photographs. This software can be used by dental students before entering radiology department, to simplify the understanding of basic principles of dental radiology. The software is presented in the website: http://hsoft.ir80.com as *Review of Dental Radiology Basics Software* and can be downloaded freely. Future revised forms also will be available.

We hope that this software can help dental students to become familiar with some Far-fetched principles of radiology and acts as a starting point for developing E-learning in radiology department and other parts of our dental schools.

